# Scrodentoid A Inhibits Mast Cell–Mediated Allergic Response by Blocking the Lyn–FcεRIβ Interaction

**DOI:** 10.3389/fimmu.2019.01103

**Published:** 2019-05-16

**Authors:** Fei Qian, Liuqiang Zhang, Shaodong Lu, Gaohui Mao, Fujiang Guo, Ping Liu, Jinwen Xu, Yiming Li

**Affiliations:** ^1^Institute of Interdisciplinary Integrative Medicine Research, Shanghai University of Traditional Chinese Medicine, Shanghai, China; ^2^School of Pharmacy, Shanghai University of Traditional Chinese Medicine, Shanghai, China

**Keywords:** antiallergy, FcεRIβ, Lyn, mast cell, scrodentoid A

## Abstract

**Background:** Mast cells are considered an attractive therapeutic target for treating allergic diseases, and the Lyn–FcεRIβ interaction is essential for mast cell activation. This study investigated the antiallergic effect of scrodentoid A (SA) on mast cells and mast cell–mediated anaphylaxis.

**Methods:** For *in vitro* experiments, mast cells were treated with SA. Cell proliferation was tested using the XTT assay. The mRNA expression of various cytokines and chemokines was measured using qPCR. The levels of histamine, eicosanoids (PGD_2_, LTC_4_), and cytokines were measured using enzyme immunoassay kits. Signaling was investigated using Western blotting and immunoprecipitation. For *in vivo* experiments, the antiallergic activity of SA was evaluated using two mouse models of passive anaphylaxis as passive cutaneous and systemic anaphylaxis. The mechanism was investigated through immunohistochemistry and immunofluorescence.

**Results:** SA considerably inhibited immunoglobulin (Ig) E-mediated mast cell activation, including β-hexosaminidase release, mRNA and protein expression of various cytokines, and PGD_2_ and LTC_4_ release_._ Oral administration of SA effectively and dose-dependently suppressed mast cell–mediated passive cutaneous and systemic anaphylaxis. SA significantly attenuated the activation of Lyn, Syk, LAT, PLCγ, JNK, Erk1/2, and Ca^2+^ mobilization without Fyn, Akt, and P38 activation by blocking the Lyn–FcεRIβ interaction.

**Conclusions:** SA suppresses mast cell–mediated allergic response by blocking the Lyn–FcεRIβ interaction *in vitro* and *in vivo*. SA may be a promising therapeutic agent for allergic and other mast cell–related diseases.

## Introduction

Allergy prevalence has been increasing worldwide, which has contributed directly or indirectly to health and economic burdens (1, 2). Mast cells are central players in both the development and maintenance of allergic diseases. Mast cell activation releases various mediators, including preformed granule-associated chemical mediators, lipid mediators, and *de novo* synthesized cytokines, which are essential in allergies (3). Immunoglobulin (Ig) E and the high-affinity receptor FcεRI are vital in mast cell activation in an allergy context (4). FcεRI, present on mast cell surface, is a tetrameric complex comprising an IgE-binding α, signal-modulating β, and two signal-transducing γ subunits. The signaling cascades elicited by FcεRI aggregation start with Lyn phosphorylation, which transphosphylate the immunoreceptor tyrosine-based activation motif (ITAM) within FcεRIβ and FcεRIγ. Subsequently, another tyrosine kinase, Syk, is recruited and binds to phosphorylated ITAM, resulting in the phosphorylation of the adaptor proteins (LAT) and phospholipase Cγ (PLCγ). The principal axis pathway is then initated, activating the downstream signaling pathways, including the mitogen-activated protein kinase (MAPK), protein kinase C (PKC), and calcium flux pathways (5–7).

Binding of FcεRIα to the allergen–IgE complex initiates mast cell activation. FcεRIβ accelerates FcεRIα maturation in mast cells, thereby increasing FcεRI receptor expression on the cell membrane (8). FcεRIβ also amplifies the cell activation signal by enhancing the FcεRIγ signal by five to seven times, accelerating mast cell activation (9). Lyn is critical for ITAM phosphorylation on FcεRIβ (10), and a weak Lyn–FcεRIβ interaction is noted before FcεRI aggregation (11–13). Lyn next binds to the phosphorylated Y219 site of ITAM within FcεRIβ, and the interaction between Lyn and FcεRI increases considerably (14, 15). The Lyn–FcεRIβ interaction is essential for human mast cell activation (16). Thus, we hypothesized that blocking the Lyn–FcεRIβ interaction may be a new direction for allergic disease treatment.

In our previous studies, five 19(4 → 3)-abeo-abietane diterpenoids (scrodentoids A-E) were firstly isolated from the whole plant of Scrophularia dentata. Among them, Scrodentoid B (SB) was considered as a potential immunosuppressive agent (17), and the other biological activities of these compounds have not been reported. Recently, it was suggested that some diterpenoid compounds have antiallergic activity, particularly in mast cell–mediated allergies (18, 19). In our further anti-allergic screening, only Scrodentoid A (SA) could modify IgE/Ag-stimulated mast cell activation and mast cell mediated anaphylaxis *in vitro*. However, the content of SA in S. dentata is very low, and its mechanism of action as an anti-allergic compound remains unknown.

This is the first study to describe the chemical conversion from SB to SA and the inhibitory effects of SA on mast cell activation, evidenced by suppressed degranulation, lipid mediator and cytokine production, and reduced downstream signaling pathway activation. In agreement with this, SA considerably inhibits mast cell–mediated anaphylactic response by blocking the Lyn–FcεRIβ interaction. Therefore, SA might serve as a novel therapeutic target for allergic diseases.

## Materials and Methods

### Reagents

Whole plants of Scrophularia dentata were collected from Tibet, China in October 2015. The plant was identified by Professor Zhili Zhao of School of Pharmacy, Shanghai University of Traditional Chinese Medicine. A voucher sample (No. CX2015) was deposited with the Herbarium of the Department of TCM Chemistry, School of Pharmacy of Shanghai University of Traditional Chinese Medicine (Shanghai, China).

ESIMS was obtained on a Bruker Daltonics Esquire 3000 plus (Bruker Daltonics, Bremen, Germany). The NMR spectra were recorded on a Bruker AM-400 spectrometer at 400 MHz for ^1^H and 100 MHz for ^13^C in CDCl_3_ Bruker AVANCE-III instrument operating at 600 MHz for ^1^H and 150 MHz for ^13^C. Silica gel (200 mesh to 300 mesh, Qingdao Haiyang Chemical Co., Ltd., Qingdao, China), C_18_ reversed-phase silica gel (150 to 200 mesh, Fuji Silysia Chemical, Ltd., Aichi, Japan) were used for column chromatography (CC). Semipreparative High-performance liquid chromatography (HPLC) was performed on an Angilent 1200 HPLC apparatus with an Eclipse XDB-C_18_ column (250 × 9.4 mm, 5 μm). All solvents used for CC were of analytical grade (Sinopharm Chemical Reagent Co., Ltd), and all solvents used for HPLC were of HPLC grade.

Mouse anti-dinitrophenyl (DNP) IgE, cell proliferation Kit II, glycine, 4-Nitrophenyl N-acetyl-β-D-glucosaminide (p-NAG), BSA, Evans Blue, Citric acid monohydrate, DMSO, *o*-phthaldialdehyde, and Ketotifen were purchased from Sigma-Aldrich (ST. Louis, MO, USA). The antibodies specific for phospho (p)-Syk (Tyr525/526), Syk, p-PLCγ (Tyr783), p-AMPK (Thr172), p-AMPK (Ser485/491), AMPK, p-P38 (Thr180/Tyr182), P38, p-JNK (Thr183/Tyr185), JNK, p-Erk1/2 (Thr202/Tyr204), Erk1/2, p-cPLA_2_ (Ser505), p-Akt (Ser473), Akt, Phospho-Tyrosine (P-Tyr-1000), Normal rabbit IgG were from Cell Signaling Technology (Danvers, MA, USA). The antibodies specific for p-Lyn (Tyr 507), Lyn, p-Fyn (Y530), Fyn, p-LAT (Y191), LAT were from Abcam China. The antibodies specific for PLCγ, FcεRIβ, FcεRIγ, GAPDH, Goat anti-rabbit-IgG HRP, Goat anti-mouse-IgG HRP were from Santa Cruz Biotechnology (Dallas, Texas, USA). Fluo-4 NW calcium assay kit, Fetal Bovine Serum (FBS), mouse IL-6 ELISA kit, mouse TNF alpha ELISA kit, Trizol Reagent, and the High-Capacity cDNA Reverse Transcription Kit were from Thermo Fisher. DNP-HSA was from Biosearch technology. Recombinatant Mouse IL-3 and recombinatant mouse SCF/c-kit Ligand were from RD systems China. LTC_4_, PGD_2_ ELISA Kit were from Cayman Chemicals (Ann Arbor, MI, USA). ECL Immobilon Western Cheniluminescent HRP Substrate was from Millipore (Millipore. Billerica, USA).

### Preparation of SA

Extracts from the dried whole plant of *S. dentata* (8 kg) were obtained with 95% EtOH (3×, each 80 L), using a reflux apparatus for 1.5 h. The extract (1,080 g) was obtained after in vacuo removal of the solvent. The extract was suspended in water and sequentially extracted using CH_2_Cl_2_, The CH_2_Cl_2_ extract was evaporated to dryness in vacuo, and the resultant CH_2_Cl_2_ fraction (243.6 g) was subjected to silica gel column chromatography (CC), eluted with EtOAc in petroleumether (PE) (0–100%, stepwise) to yield 12 fractions (Fr. 1–Fr. 12). Fr. 5 was separated repeatedly using CC and was further separated through reversed-phase HPLC by using CH_3_CN-H_2_O (83:17) to yield SA (17) (**1**, 21 mg). Fr. 7 was separated repeatedly using CC and was further separated through ODS CC with an MeOH gradient (80–100%) in H_2_O to yield SB (17) (**2**, 367 mg).

Trifluoroacetic acid (0.3 mL) was added into a solution of SB (300 mg, 1.007 mmol) in CH_2_Cl_2_ (20 mL), then the mixture was stirred at 25°C for 12 h. The reaction mixture was poured into ice water, adjusted to pH = 7 with NaHCO_3_ (a.q.), then the residue was extracted with CH_2_Cl_2_ (40 mL×3), dried over Na_2_SO_4_, and concentrated to give the product of compound **3** (238 mg, yield: 80%). ^1^H NMR (CDCl_3_, 400 MHz) δ 2.28 (1H, dt, *J* = 13.1/3.1 Hz, H-1a), 1.75 (1H, td, *J* = 13.1/4.0 Hz, H-1b), 1.87 (1H, dq, *J* = 13.0/3.1 Hz, H-2a), 1.40 (1H, qd, *J* = 13.0/4.0 Hz, H-2b), 2.06 (1H, m, H-3), 2.71 (1H, m, H-5), 2.64 (1H, m, H-6a), 2.76 (1H,m, H-6b), 6.96 (1H, s, H-11), 7.93 (1H, s, H-14), 5.15 (1H, s, H-16a), 5.44 (1H, s, H-16b), 2.13 (3H, s, H-17), 4.93 (1H, s, H-18a), 4.70 (1H, s, H-18b), 1.12 (3H, d, *J* = 6.5 Hz, H-19), 1.07 (3H, s, H-20). ESIMS *m/z* 295 [M-H]^−^.

To a solution of compound **3** (200 mg) in ethyl acetate (40 mL), was added Pd/C (50 mg) and the mixture was stirred under H_2_ for 0.5 h. The precipitate was removed by filtration through Celite, and the filtrate was evaporated under reduced pressure. The residue was chromatographed on a silica gel (PE:EtOAc = 8:1) to give SA (200 mg) as a white amorphous powder in quantitative yield. ^1^H NMR (CDCl_3_, 400 MHz) δ 2.20 (1H, dt, *J* = 13.2/3.0 Hz, H-1a), 1.72 (1H, td, *J* = 13.2/4.0 Hz, H-1b), 1.84 (1H, dq, *J* = 13.0/3.0 Hz, H-2a), 1.37 (1H, qd, *J* = 13.0/4.0 Hz, H-2b), 2.05 (1H, m, H-3), 2.66 (1H, m, H-5), 2.60 (1H, m, H-6a), 2.71 (1H,m, H-6b), 6.77 (1H, s, H-11), 7.96 (1H, s, H-14), 3.15 (1H, seq, *J* = 6.8 Hz, H-15), 1.24 (3H, d, *J* = 6.8 Hz, H-16), 1.25 (3H, d, *J* = 6.8 Hz, H-17), 4.89 (1H, s, H-18a), 4.67 (1H, s, H-18b), 1.09 (3H, d, *J* = 6.5 Hz, H-19), 1.02 (3H, s, H-20). ESIMS *m/z* 297 [M-H]^−^.

### BMMC Culture

BMMCs were derived from the bone marrow of Balb/C mice. Cells were maintained in RPMI-1640, 50 μmol/L 2-mercaptoethanol, 1 mmol/L sodium pyruvate, 100 U/mL penicillin−100 μg/mL streptomycin, 0.1 mmol/L non-essential amino acids, 25 mmol/L HEPES, 10% FBS, and 10 ng/mL mouse recombinant IL-3 and SCF. Cells were fed every 7 days. After 4 weeks, cell purity was determined by measuring CD117 and FcεRI expression through flow cytometry: 95% of cells were positive for both CD117 and FcεRI.

### Viability Analysis Using XTT Assay

BMMCs were dispensed into 96-well plates (5 × 10^4^ cells/well), and the cells were treated with SA (1, 10, 25, and 50 μmol/L) for 24 h. Cell viability was evaluated using a XTT-based cell proliferation assay kit, according to the manufacturer's protocol. Cell viability was expressed as the percentage OD_test_/OD_control_, where OD_test_ and OD_control_ indicate the optical densities of cells exposed to the test and control compounds, respectively.

### Degranulation Assay

BMMCs were first incubated at 5 × 10^5^ cells/mL with 0.5 μg/mL anti-DNP-IgE in complete media overnight for sensitization. Cells were resuspended in HEPES buffer (10 mM HEPES, 137 mM NaCl, 2.7 mM KCl, 0.4 mM Na_2_HPO_4_·7H_2_O, 5.6 mM glucose, 1.8 mM CaCl_2_·2H_2_O, 1.3 mM MgSO_4_·7H_2_O; Sigma-Aldrich) and transferred to a round-bottom 96-well plate. Then, cells were treated with SA for 30 min. Finally, cells were treated with 0.1 μg/mL DNP-HSA for 30 min for stimulation. Mast cell degranulation was then assessed by measuring β-hexosaminidase (β-hex) release.

RBL-2H3 cells were dispensed into a 96-well plate and incubated for 5 h to allow cells to adhere. The cells were sensitized with 0.1 μg/mL anti-DNP-IgE for 20 h. The next day, the medium was discarded and the cells were washed two times with HEPES buffer. The cells were then treated with SA for 30 min, followed by stimulation with DNP-HSA for 30 min. Finally, mast cell degranulation was assessed by measuring β-hex release.

β-hex in the supernatants and pellet was quantified theough the hydrolysis of p-NAG in 0.1 M sodium citrate buffer (pH 4.5) for 90 min at 37°C. After adding 50 μL glycine (pH 10.7) to each fraction, the absorbance was measured at A_405_ and A_570_ in a microplate reader. The amount of total β-hex released was calculated using the following formula: % release = 100 × supernatant absorbance/(0.5 × supernatant absorbance + pellet absorbance).

### Histamine Assay

IgE-sensitized BMMCs were washed, resuspended in HEPES at 2.5 × 10^6^ cells/mL, and incubated with SA for 30 min at 37°C and 5% CO_2_. The BMMCs were then stimulated using 0.1 μg/mL DNP-HSA for 30 min at 37°C and 5% CO_2_. Samples were centrifuged at 300 × *g* for 5 min to obtain the supernatant. Histamine (working standards of 7.8–500 ng/mL) was freshly prepared. Histamine standards and supernatants were transferred to a 96-well flat-bottom plate and mixed with 12 μL of 1 M NaOH and 3 μL of 10 mg/mL *o*-phthalaldehyde. After 4 min at room temperature, 6 μL of 3 M HCl was added to stop the reaction. The fluorescence intensity was read using a 360-nm excitation filter and a 450-nm emission filter.

### RT-PCR Analysis

IgE-sensitized BMMCs or RBL-2H3 cells treatment with SA for 30 min stimulated with DNP-HSA for 1 h, total RNA was extracted using Trizol Reagent followed the manufacturer's instructions. Complementary DNA (cDNA) was then synthesized from 1 μg of total RNA using the High-Capacity cDNA Reverse Transcription Kit. Real-time PCR was performed using SYBR Green Supermix with sequence-specific primers in the 7500 Fast Real-Time PCR System. The GAPDH mRNA expression was used as an internal control. The following sequence-specific primers were used in this study: rat IL-4 (Forward 5′ to 3′: GAGGACCAGAACGAGACA; Reverse 5′ to 3′: GTGGAAGAGCATCAGGAG), rat CCL2 (Forward 5′ to 3′: GCAGAGACACAGACAGAGG; Reverse 5′ to 3′: CCAGAAGCGTGACAGAGA), rat TNF-α (Forward 5′ to 3′: CCCTGTTCTGCTTTCTCA; Reverse 5′ to 3′: GTTCTCCGTGGTGTTCCT), rat IL-13 (Forward 5′ to 3′: AGCAACATCACACAAGACC; Reverse 5′ to 3′: GGTTACAGAGGCCATTCA), rat GAPDH (Forward 5′ to 3′: GGCACAGTCAAGGCTGAGAATG; Reverse 5′ to 3′: ATGGTGGTGAAGACGCCAGTA), mouse IL-3 (Forward 5′ to 3′: AGGGTCCTTCATCATCAGTTTT; Reverse 5′ to 3′: GCTCTACCACCAGCATCCACA), mouse IL-5 (Forward 5′ to 3′: GGCTTCCTGCTCCTATCTAACTTCA; Reverse 5′ to 3′: ACAGTCATGGCACAGTCTGATTCAT), mouse IL-13 (Forward 5′ to 3′: CTCTTGCTTGCCTTGGTGGTC; Reverse 5′ to 3′: AGGGGAGTCTGGTCTTGTGTGAT), mouse IL-33 (Forward 5′ to 3′: CGGAGTAGTCCTTGTCGTTGG; Reverse 5′ to 3′: GGATGGGAAGAAGCTGATGGT), mouse IL-1β (Forward 5′ to 3′: TGTGTTTTCCTCCTTGCCTCTGAT; Reverse 5′ to 3′: TGCTGCCTAATGTCCCCTTGAAT), mouse IL-6 (Forward 5′ to 3′: TCACAGAAGGAGTGGCTAAGGACC; Reverse 5′ to 3′: ACGCACTAGGTTTGCCGAGTAGAT), mouse GM-SF (Forward 5′ to 3′: CACAAGTTACCACCTATGCGGA; Reverse 5′ to 3′: GAGTTCCTGGCTCATTACGCA), mouse MIP-1a (Forward 5′ to 3′: GCTCCCAGCCAGGTGTCATTTT; Reverse 5′ to 3′: AAGACTCTCAGGCATTCAGTTCCAG), mouse RANTES (Forward 5′ to 3′: GTGTGCCAACCCAGAGAAGAAGT; Reverse 5′ to 3′: AGCAAGCAATGACAGGGAAGCT), mouse GAPDH (Forward 5′ to 3′: AAGAAGGTGGTGAAGCAGG; Reverse 5′ to 3′: GAAGGTGGAAGAGTGGGAGT).

### Cytokine Enzyme-Linked Immunosorbent Assay (ELISA)

BMMCs were dispensed into 24-well plates (2.5 × 10^5^ cells per well) and sensitized with 0.5 μg/mL anti-DNP-IgE for 20 h. SA was dissolved in a medium containing DMSO and added to the cells to a final concentration of 5, 10, and 25 μmol/L for 30 min. Then cells were stimulated with DNP-HSA for 24 h for TNF, IL-6, and IL-13 or 6 h for LTC_4_ and PGD_2_.

The supernatant was collected, centrifuged at 300 × *g* for 5 min to remove cell debris, and stored at −80°C for ELISA. The supernatant was analyzed for TNF, IL-6, and IL-13 release using ELISA kits and for LTC_4_ and PGD_2_ with an ELISA kit, according to the manufacturer's protocol.

### Intracellular Ca^2+^ Mobilization

BMMC Ca^2+^ mobilization was measured using a Fluo-4 NW Calcium Assay kit. IgE-sensitized BMMCs were washed and resuspended in assay buffer at 2.5 × 10^6^ cells/mL. A 50 μL suspension was transferred to a 96-well plate, loaded with dye for 30 min at 37°C, and treated with various concentrations of SA and PP2 for 30 min at room temperature. Fluorescence was read at 485-nm excitation and 516-nm emission filters. DNP-HSA (0.1 μg/mL) was added at 100 s, and calcium responses were recorded simultaneously.

### Western Blotting

IgE-sensitized BMMCs were washed, resuspended in HEPES buffer at 5 × 10^7^ cells/mL, treated with SA (5, 10, and 25 μmol/L) or PP2 (10 μmol/L) for 30 min, and stimulated with 0.1 μg/mL DNP-HSA for 5 or 15 min at 37°C and 5% CO_2_. Or non-stimulated BMMCs (5 × 10^7^ cells/mL) were treated with SA (5, 10, and 25 μmol/L) for 30 min. The reactions were terminated by adding a lysis buffer containing 1% Triton X-100, 1 × complete protease inhibitor cocktail, 50 μg/mL 3,4-dichloroisocoumarim (Roche Molecular Biochemicals, Indianapolis, Indiana, USA), 1 × protease inhibitor cocktail, 1 mM benzamidine, 1 mM sodium orthovanadate, 5.4 mM sodium pyrophosphate, and 50 mM sodium fluoride. Samples were boiled at 95°C for 5 min in 1 × NuPAGE sample buffer (Life Technologies). After measuring the protein concentration using the Bradford protein assay, samples were adjusted to the same concentration.

Cell protein samples were separated on a NuPAGE Novex 4–12% Bis-Tris gel under non-reduced condition. The proteins were then transferred to a nitrocellulose membrane at 20 V for 1 h. The membrane was blocked with 4% BSA-TBST for 1 h and probed with appropriate antibodies overnight at 4°C. The membrane was washed three times with TBST and incubated with HRP-conjugated goat anti-rabbit IgG or HRP-conjugated goat anti-mouse IgG diluted with 4% BSA-TBST for 1 h at room temperature. Finally, the membrane was developed with a chemiluminescent peroxidase substrate for 1 min and exposed to chemiluminescence film.

### Immunoprecipitation

IgE-sensitized BMMCs were washed, resuspended in HEPES buffer at 5 × 10^7^ cells/mL, treated with or without SA (25 μmol/L) for 30 min, and stimulated with DNP-HSA (0.1 μg/mL) for 10 min at 37°C and 5% CO_2_. Or non-stimulated BMMCs (5 × 10^7^ cells/mL) were treated with SA (5, 10, and 25 μmol/L) for 30 min. The reactions were terminated by adding the lysis buffer. Samples were put on ice for 30 min, span at 12,000 rpm, 15 min to get the soluble lysates. The soluble lysates were immunoprecipitated with the appropriate antibodies for 2 h at 4°C. Protein A/G Sepharose beads (Santa Cruz Biotechnology) were then added, and the samples were incubated overnight at 4°C. After washing three times, the samples were boiled at 95°C for 5 min in 1 × NuPAGE sample buffer. Then, the samples were resolved on SDS-PAGE gels (10%) under non-reduced condition and blotted as described.

### IgE-Mediated Anaphylaxis in Mice

Six-week-old male ICR mice and Balb/c mice were obtained from the B&K Laboratory Animal Corp. Ltd. (Shanghai, China). All animal experiments were performed according to the Health Guidelines of the Shanghai University of Traditional Chinese Medicine, China, and protocols were approved by the Institutional Animal Ethics Committee of Shanghai University of Traditional Chinese Medicine (SZY201710088).

### Passive Cutaneous Anaphylaxis

Passive cutaneous anaphylaxis (PCA) was performed as described previously (9). In brief, 1 μg of anti-DNP-IgE was intradermally injected into the right ear of 7-week-old male mice. The next day, the mice received oral administration of 25, 50, or 100 mg/kg SA or 50 mg/kg ketotifen (Sigma-Aldrich). After 30 min, the mice were challenged for 30 min by intravenous injection of 100 μg DNP-HSA in 300 μL saline containing 0.5% Evans blue. Finally, Evans blue was extracted after 24 h at room temperature with 300 μL of formamide and measured by absorbance at 630 nm.

### Passive Systemic Anaphylaxis

Seven-week-old male mice were sensitized by intravenous injection of 2 μg of anti-DNP-IgE for 24 h. The next day, the mice received oral administration of 25, 50 mg/kg SA or 50 mg/kg ketotifen. After 30 min, the mice were challenged by intravenous injection of 1 mg of DNP-HSA. After antigen challenge, rectal temperature was measured and recorded every 5 min for 50 min with a digital thermometer. Mice were sacrificed 1 h after antigen challenge. Blood serum was collected. Ear tissues were fixed with 4% paraformaldehyde and embedded in paraffin. Sections were stained with H&E, toluidine blue. For immunofluorescence (Lyn and FcεRIβ), mice were sacrificed 10 min after antigen challenge. Briefly, sections were incubated overnight with anti-Lyn and anti-FcεRIβ. Staining was detected using the appropriated secondary antibody. Nuclei were stained with DAPI. The stained sections were scanned using panoramic slide scanner (3D HISTECH, Hungary). The images were calculated using Image-Pro plus 6.0.

### Statistical Analysis

Experiments were conducted in triplicate, and data are presented as mean (*n* = 3) ± standard error of the mean (SEM). *P* values were determined using one-way ANOVA using GraphPad Prism 6.0. Statistical significance was set at *P* of ≤ 0.05, ≤ 0.01, or ≤ 0.001.

## Results

### Preparation of SA

**Figure 1:**
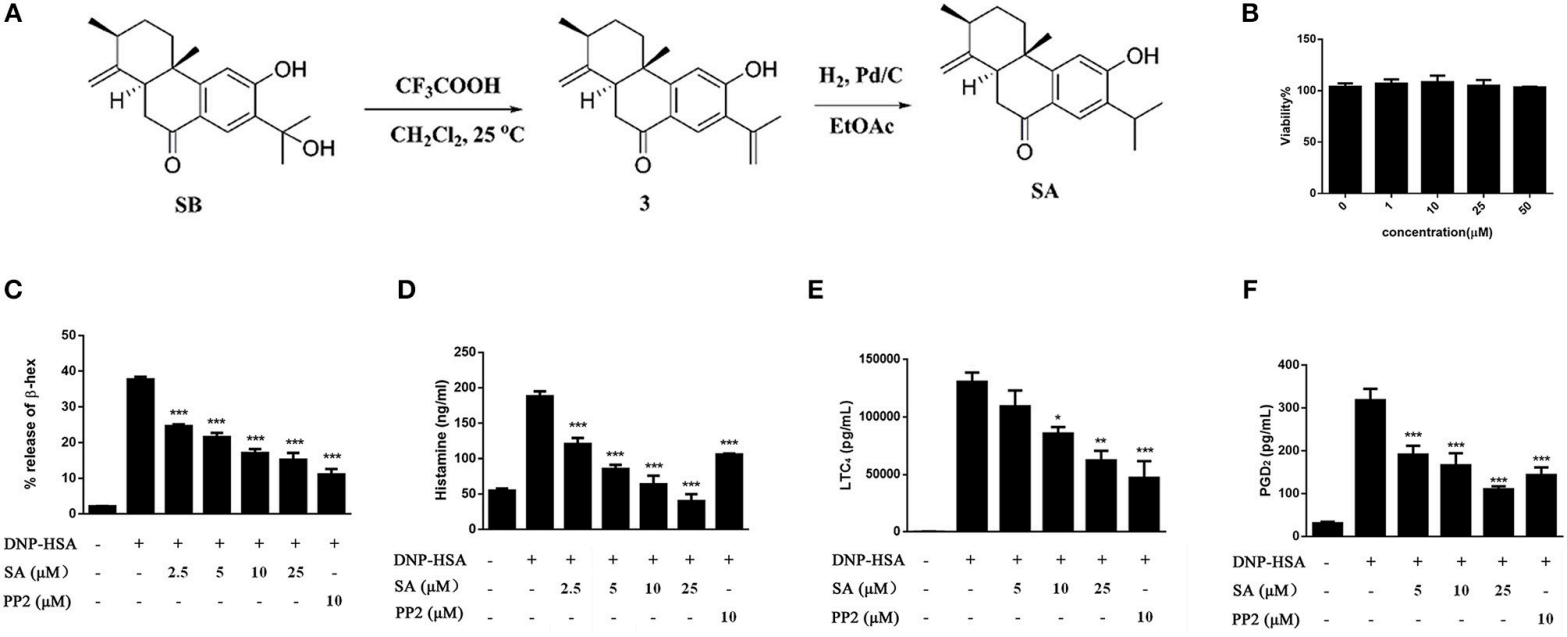
The preparation of SA and the effect of SA on viability and degranulation of mast cells. **(A)** A schematic diagram for the preparation of SA through chemical transformation. **(B)** BMMCs were incubated in the presence of 1, 10, 25, or 50 μmol/L of SA for 24 h. Cell viability was assayed using an XTT assay. **(C–F)** BMMCs were sensitized with anti-DNP IgE with or without 5, 10, or 25 μmol/L of SA for 30 min, challenged with DNP-HSA-induced release of β-hex **(C)** and histamine **(D)** at 30 min, and secretion of LTC_4_ **(E)** and PGD_2_ **(F)** at 6 h was assessed. The results are expressed as mean ± SEM from three individual experiments. *P < 0.05, **P < 0.01, ***P < 0.001 vs. the IgE/Ag-treated group.

In order to enrich enough SA to meet the needs of pharmacological experiments, we obtained a total of 200 mg of SA through extraction, separation and chemical transformation (Figure 1A).

### SA Inhibits Degranulation and Lipid Mediators in IgE/Ag-Stimulated BMMCs

Before investigating the effect of SA (Figure 1A) on mast cell activation, we examined the cytotoxicity of SA on BMMCs by using XTT assay. BMMCs were treatmed with various concentrations (1, 10, 25, and 50 μmol/L) of SA for 24 h (Figure 1B); SA did not exhibit any cytotoxicity, even at 50 μmol/L. Therefore, we used SA at concentrations of up to 25 μmol/L for all *in vitro* experiments. To determine the effect of SA on BMMC degranulation, we first sensitized BMMCs with anti-DNP-IgE for 20 h and then incubated cells with various concentrations (2.5, 5, 10, and 25 μmol/L) of SA. After 30 min, BMMCs were stimulated with DNP-HSA for 30 min, and then mast cells released β-hex and histamine. SA treatment significantly decreased β-hex (Figure 1C) and histamine (Figure 1D) release in a dose-dependent manner. Furthermore, the inhibitory effect of SA on histamine was better than that obtained by PP2, a tyrosine kinase inhibitor.

FcεRI-mediated mast cells also release lipid mediators after activation—prostaglandin D_2_ (PGD_2_) and LTC_4_. To further clarify the effect of SA, we sensitized BMMCs with anti-DNP-IgE, followed by incubation with or without SA for 30 min. The cells were then activated with DNP-HSA for 6 h. When BMMCs were activated for 6 h, cells released a large amount of LTC_4_ (Figure 1E), but SA treatment significantly decreased LTC_4_ release in a dose-dependent manner. SA also considerably attenuated PGD_2_ generation after cells were stimulated with DNP-HSA for 6 h (Figure 1F).

### SA Attenuated FcεRI-Mediated Cytokine Production in BMMCs

**Figure 2:**
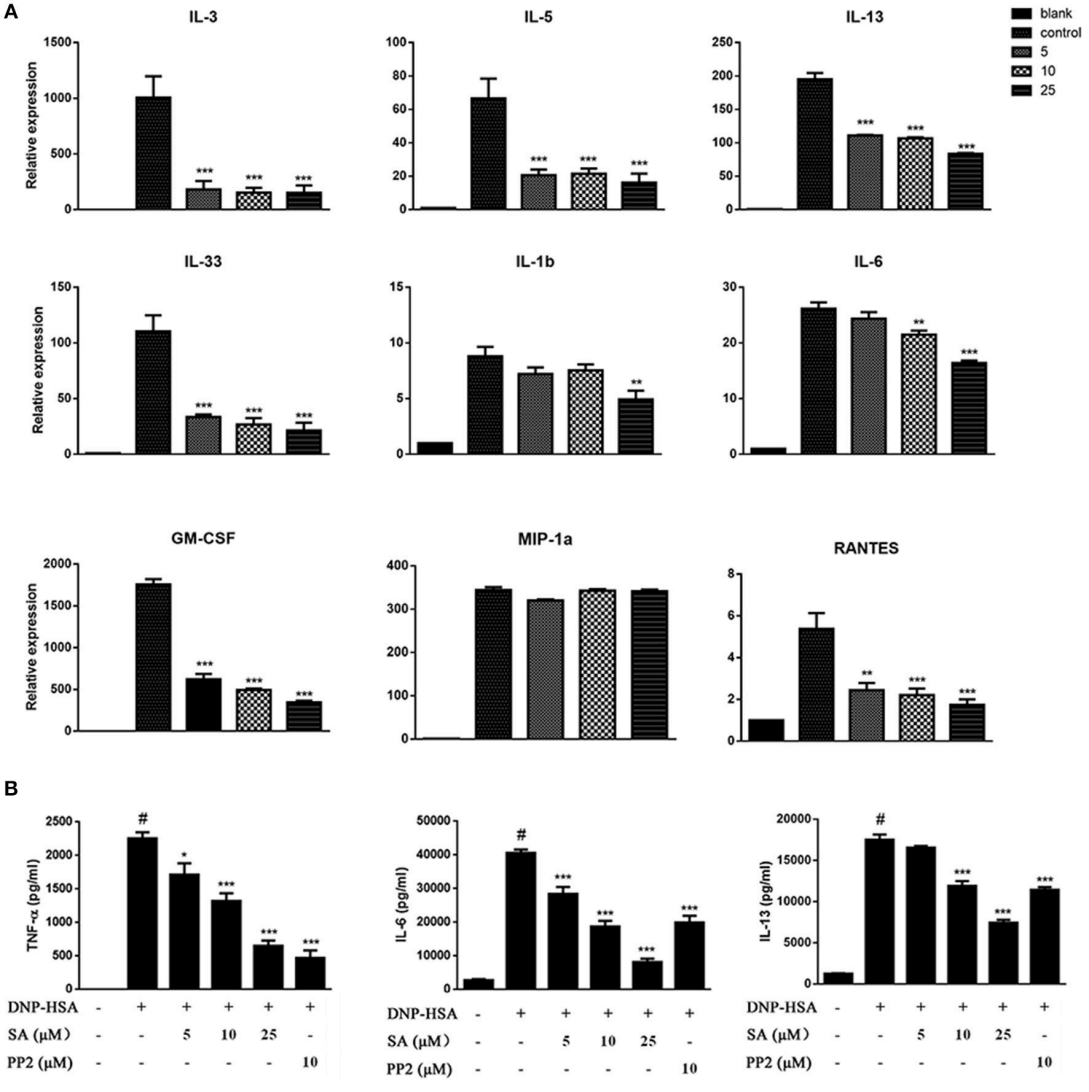
SA inhibits IgE/Ag-stimulated cytokine production. **(A)** BMMCs were sensitized with anti-DNP IgE with or without 5, 10, or 25 μmol/L of SA for 30 min and challenged with DNP-HSA for 1 h. Cytokine and chemokine mRNA expression was measured by RT-PCR. **(B)** TNF-α, IL-6, and IL-13 levels were measured by ELISA at 24 h. The results are expressed as mean ± SEM from three individual experiments. ^#^*P* < 0.05 vs. the non-stimulated mast cell group. *P < 0.05, **P < 0.01, ***P < 0.001 vs. the IgE/Ag- treated group.

Engagement of the FcεRI receptor leads to the production of multiple cytokines and chemokines essential for mast cell function and allergic response. To determine the effect of SA on FcεRI-mediated cytokines and chemokines, BMMCs were sensitized with anti-DNP-IgE overnight, and pretreatment with SA for 30 min, then stimulated with DNP-HSA for 1 h. We found that SA significantly inhibited the expression of cytokine mRNAs in FcεRI-mediated BMMCs. These include Th2-related cytokines and inflammation-related cytokines. However, the expression of MIP-1α which belongs to chemokines remained unaltered (Figure 2A). Subsequently, we used ELISA to confirm the effect of SA on FcεRI-mediated secretion of TNF-α, IL-6, and IL-13. As shown in Figure 2B, treatment with SA significantly reduced TNF-α, IL-6, and IL-13 secretion in a dose-dependent manner. In particular, SA at 25 μmol/L considerably inhibited IL-6 release, and the inhibition rate was up to 79.9%. These data indicate that SA plays a role in stabilizing mast cells, inhibiting their activation.

### Effect of SA on RBL-2H3 Cells

**Figure 3:**
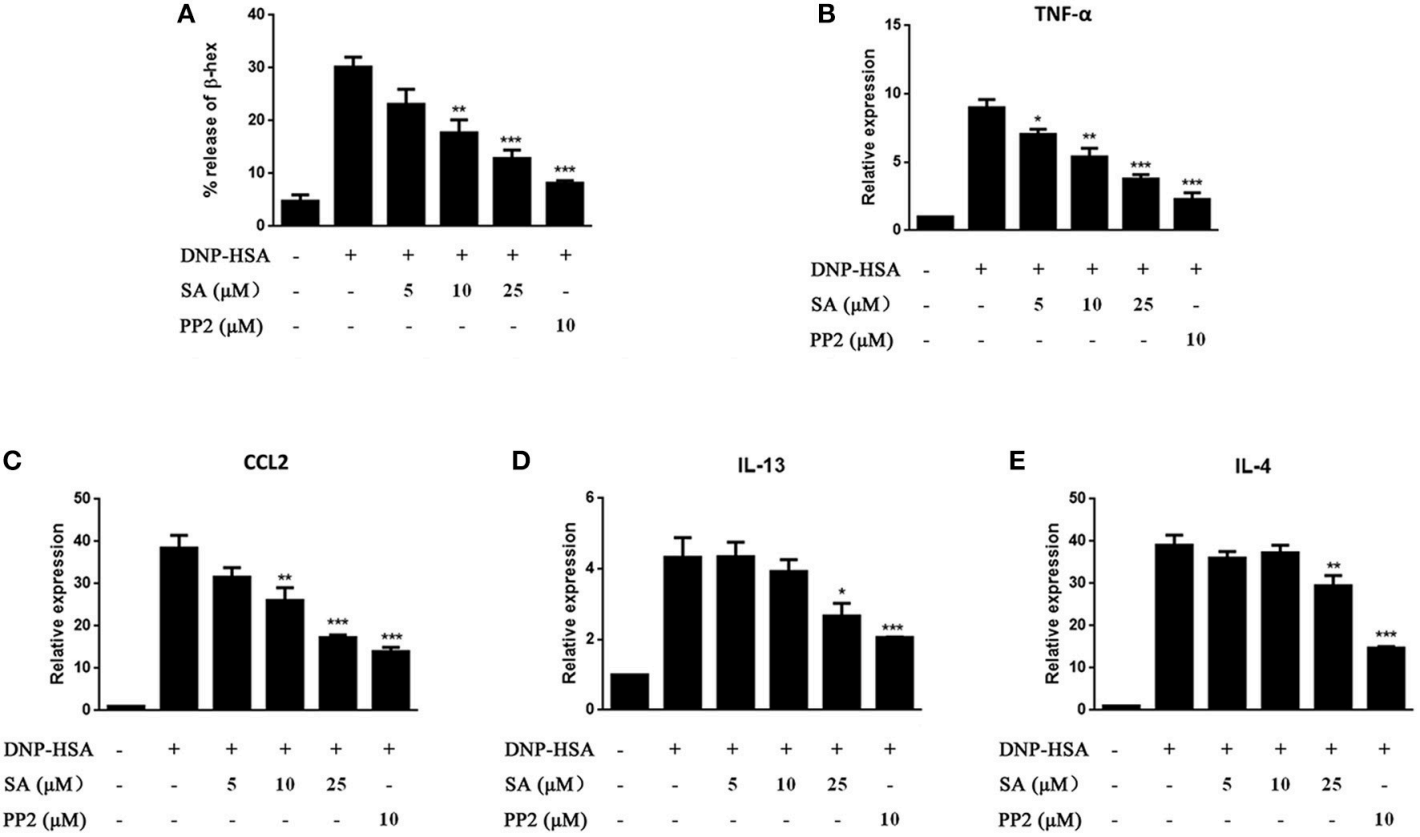
Effect of SA on IgE/Ag-stimulated RBL-2H3 cells. RBL-2H3 cells were sensitized with anti-DNP IgE with or without 5, 10, or 25 μmol/L of SA for 30 min, challenged with DNP-HSA induced release of β-hex **(A)** at 30 min, the mRNA expression of TNF-α **(B)**, CCL2 **(C)**, IL-13 **(D)**, and IL-4 **(E)** was measured by RT-PCR at 1 h. The results are expressed as mean ± SEM from three individual experiments. *P < 0.05, **P < 0.01, ***P < 0.001 vs. the IgE/Ag-treated group.

We next evaluated the effect of SA on IgE-activated RBL-2H3 cells. IgE-sensitized RBL-2H3 cells were pretreatment with SA for 30 min, then stimulated with DNP-HSA for 30 min or 1 h. After 30 min of DNP-HSA stimulation, the supernatant was assayed for β-hex release (Figure 3A), SA significantly inhibited β-hex release in a concentration-dependent manner. And SA also attenuated the mRNA expressions of TNF-α (Figure 3B), CCL2 (Figure 3C), IL-13 (Figure 3D), and IL-4 (Figure 3E) after RBL-2H3 cells were stimulated with DNP-HSA for 1 h. This further demonstrated that SA suppressed the release of allergic mediators from mast cells.

### SA Suppresses Anaphylactic Responses in Mice

**Figure 4:**
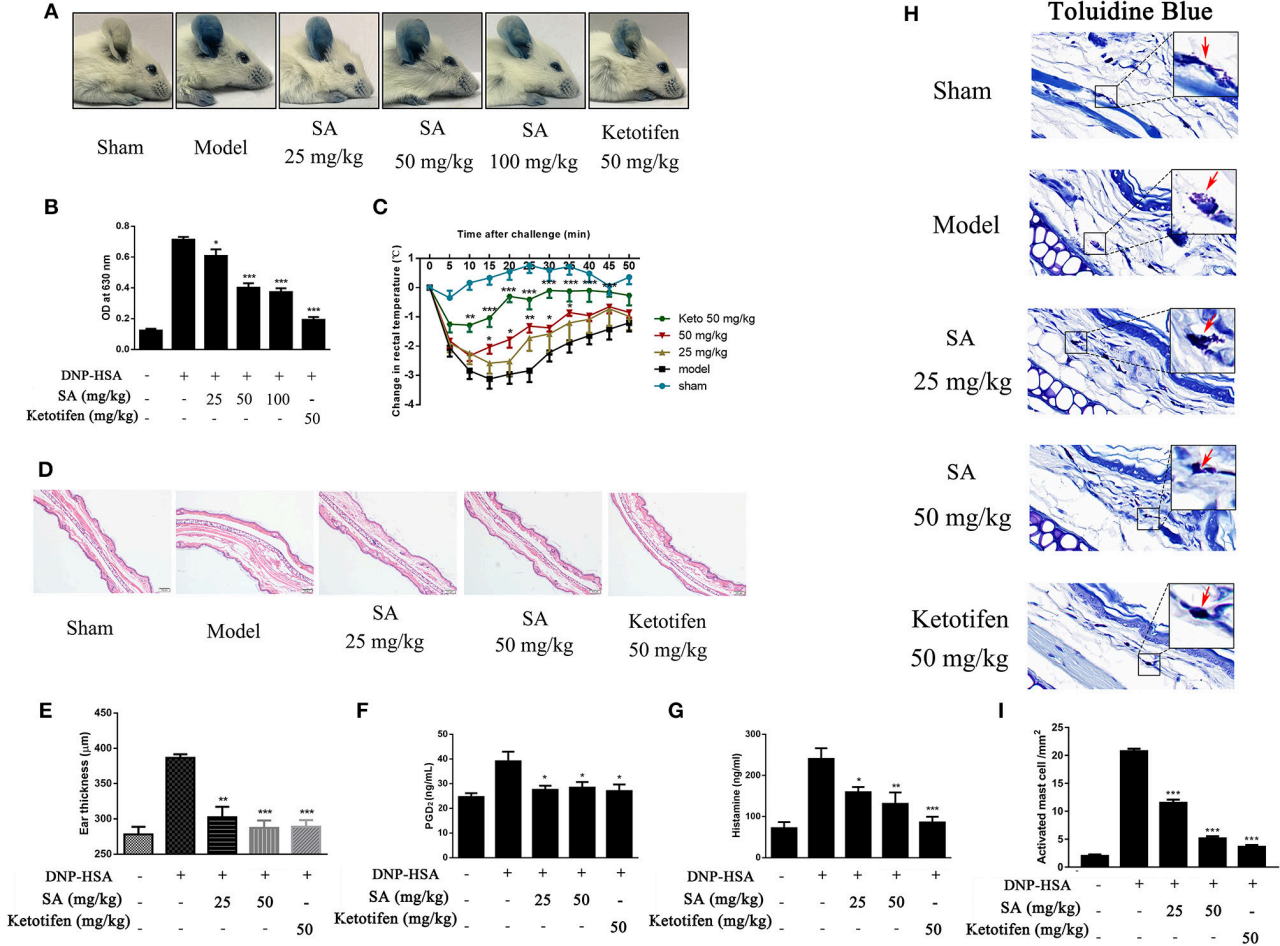
Effect of SA on IgE-mediated PCA and PSA reaction. **(A,B)** In the PCA test, ICR mice were sensitized with anti-DNP-IgE or saline for 24 h. Then, mice were treated with SA or ketotifen for 30 min, followed by intravenous injection with DNP-HSA containing 0.5% Evans blue. **(A)** Representative photos of ears showing dye extravasation. **(B)** Evans blue was extracted in formaldehyde and quantified as absorbance at 630 nm. **(C–H)** In the PSA test, mice were injected with anti-DNP-IgE (2 μg) or saline and 24 h later were orally administered SA or ketotifen; after 30 min, DNP-HSA (2 mg) was intravenously injected. Blood was collected 1 h after antigen challenge, and serum histamine and PGD_2_ were assessed. **(C)** Rectal temperatures were assessed every 5 min for 50 min. **(D)** Histological section of ears of mice, stained with HE. Bar, 100 μm. **(E)** Ear thickness was calculated based on histological section. **(F)** Serum levels of PGD_2_. **(G)** Serum levels of histamine. **(H)** Tissue mast cells in ear were assessed using toluidine blue staining. Arrowheads indicate individual mast cells. Bars, 20 μm. **(I)** Absolute numbers of activated mast cell per mm^2^ (*n* = 8 mice in each group. Data are represented as mean ± SEM, *P < 0.05, **P < 0.01, ***P < 0.001 vs. the IgE/Ag-treated mice).

Given that SA downregulated the release of allergic mediators *in vitro*, we used PCA in the ear and passive systemic anaphylaxis (PSA) to evaluate the action of SA in anaphylactic responses *in vivo*. PCA was elicited by subcutaneous injection of anti-DNP-IgE into the ear. After 24 h, mice treated with various concentrations (25, 50, and 100 mg/kg) of SA for 30 min. Then, mice received intravenous DNP-HSA solution containing 0.5% Evans blue dye. Mice challenged with DNP-HSA showed a clear PCA response in the ear. SA significantly reduced the PCA response on visual inspection (Figure 4A) and the Evans blue dye extracted from the reaction site of the ear (Figure 4B), but no difference was found between SA at 50 and 100 mg/kg. Therefore, we used SA at 25 and 50 mg/kg in the PSA assay.

PSA is an IgE-mediated type I immediate hypersensitivity reaction. Mice were sensitized with anti-DNP-IgE overnight and treated with or without SA for 30 min; then, they were intravenously injected with DNP-HSA to elicit an anaphylactic response. Rectal temperature was recorded every 5 min to monitor the magnitude of the PSA response. Mice challenged with DNP-HSA showed an evident rectal temperature drop by 10–25 min after injection. By contrast, mice treated with SA or ketotifen showed significantly reduced rectal temperature drop (Figure 4C). Histological analysis of ear sections at 1 h after injection of DNP-HSA also confirmed the antiallergic effect of SA (Figures 4D,E). In addition, SA significantly inhibited serum levels of PGD_2_ (Figure 4F) and histamine (Figure 4G). In particular, at the same concentration, SA was comparable to ketotifen, a H1 blocker and mast cell stabilizer.

Because SA inhibited mast cell activation *in vitro*, we hypothesized that SA achieves the inhibition effect of anaphylactic responses by stabilizing mast cells. Thus, we examined whether mast cell degranulation occurs or not in PSA with SA. Toluidine blue staining of ear sections showed nearly activated mast cells after allergen exposure (red arrow), whereas SA significantly reduced mast cell activation, particularly at 50 mg/kg, most mast cells were at rest status (Figure 4H). In addition, as shown as Figure 4I, SA suppressed the absolute numbers of activated mast cell. These data indicate that SA inhibits allergic response *in vivo* by suppressing mast cell activation.

### SA Inhibits Ca^2+^ Mobilization in IgE-Stimulated BMMCs

**Figure 5:**
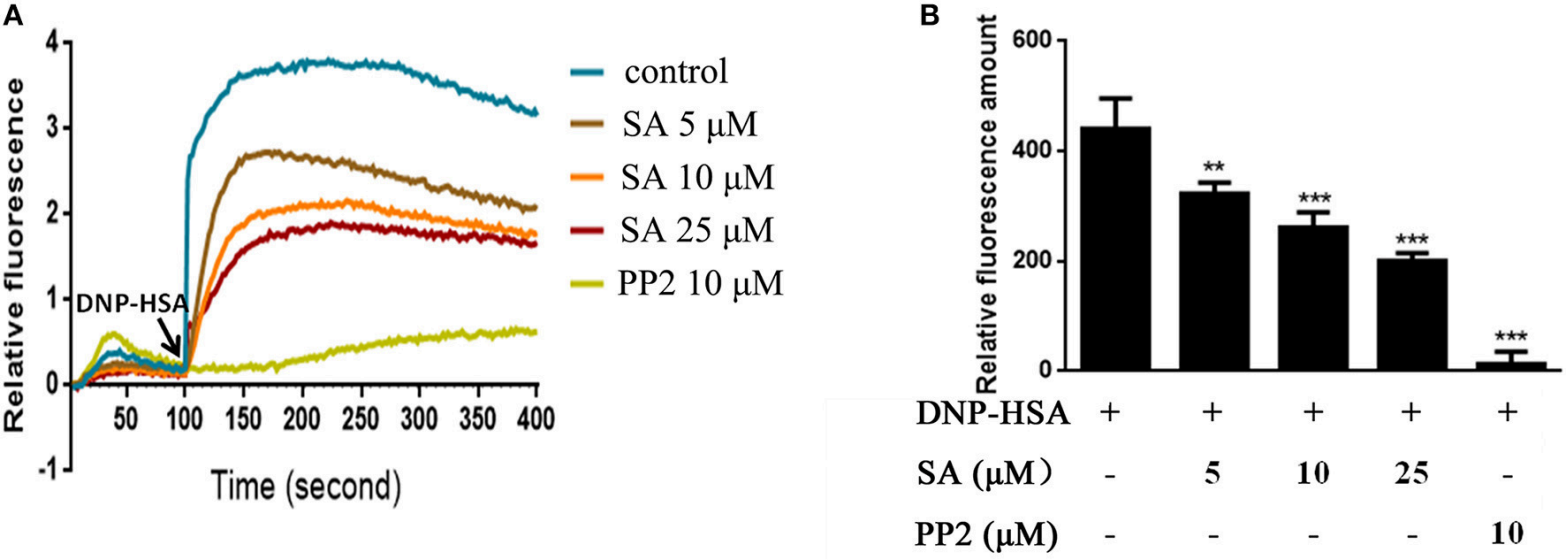
SA inhibits Ca^2+^ mobilization in IgE/Ag-stimulated BMMCs. **(A)** Calcium flux was measured by Fluo-4 NW Calcium Assay kit at the indicated times after stimulation. **(B)** The amount of calcium flux that was accumulated from BMMCs challenged DNP-HSA 0 s to 300 s. Data are represented as mean ± SEM from three experiments. **P < 0.01, ***P < 0.001 vs. the IgE/Ag-treated group.

Intracellular calcium is an important second messenger in cells, and a transient increase in intracellular calcium is crucial for the activation of mast cells (6, 20). Therefore, we explored the effect of SA on Ca^2+^ influx in BMMCs. BMMCs had high levels of calcium flux after allergen exposure and reached a peak after BMMCs challenged DNP-HSA for 50 s (Figure 5A). As expected, SA dramatically decreased intracellular calcium levels (Figure 5A), and effectively inhibited the amount of calcium levels which was accumulated from BMMCs challenged DNP-HSA 0 s to 300 s (Figure 5B).

### SA Altered FcεRI Signaling Events in BMMCs

**Figure 6:**
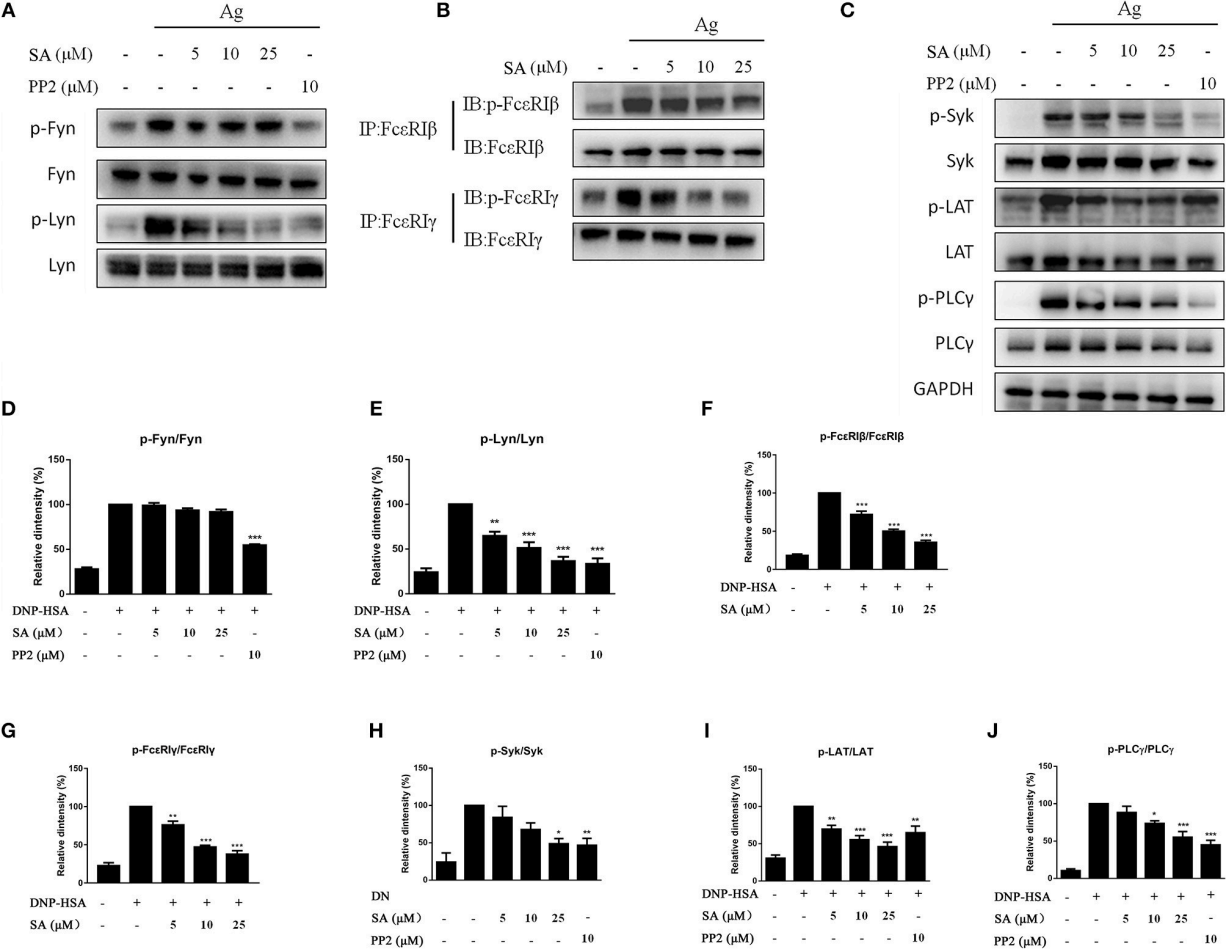
Effect of SA on FcεRI-mediated cell signaling in IgE/Ag-induced BMMCs. **(A–C)** IgE-sensitized BMMCs were treated with or without SA for 30 min and stimulated with DNP-HSA for 5 min. Then, protein lysates were analyzed for the phosphorylated and non-phosphorylated forms of Fyn, Lyn, FcεRIβ, FcεRIγ, Syk, LAT, PLCγ, using Western blotting or immunoprecipitation. **(D–J)** The related ratios of p-Fyn/Fyn **(D)**, p-Lyn/Lyn **(E)**, p-FcεRIβ/FcεRIβ **(F)**, p-FcεRIγ/FcεRIγ **(G)**, p-Syk/Syk **(H)**, p-LAT/LAT **(I)**, p-PLCγ/ PLCγ **(J)** protein levels were determined using densitometry statistical analysis. Data are represented as mean ± SEM from three experiments. *P < 0.05, **P < 0.01, ***P < 0.001 vs. the IgE/Ag-treated group.

**Figure 7:**
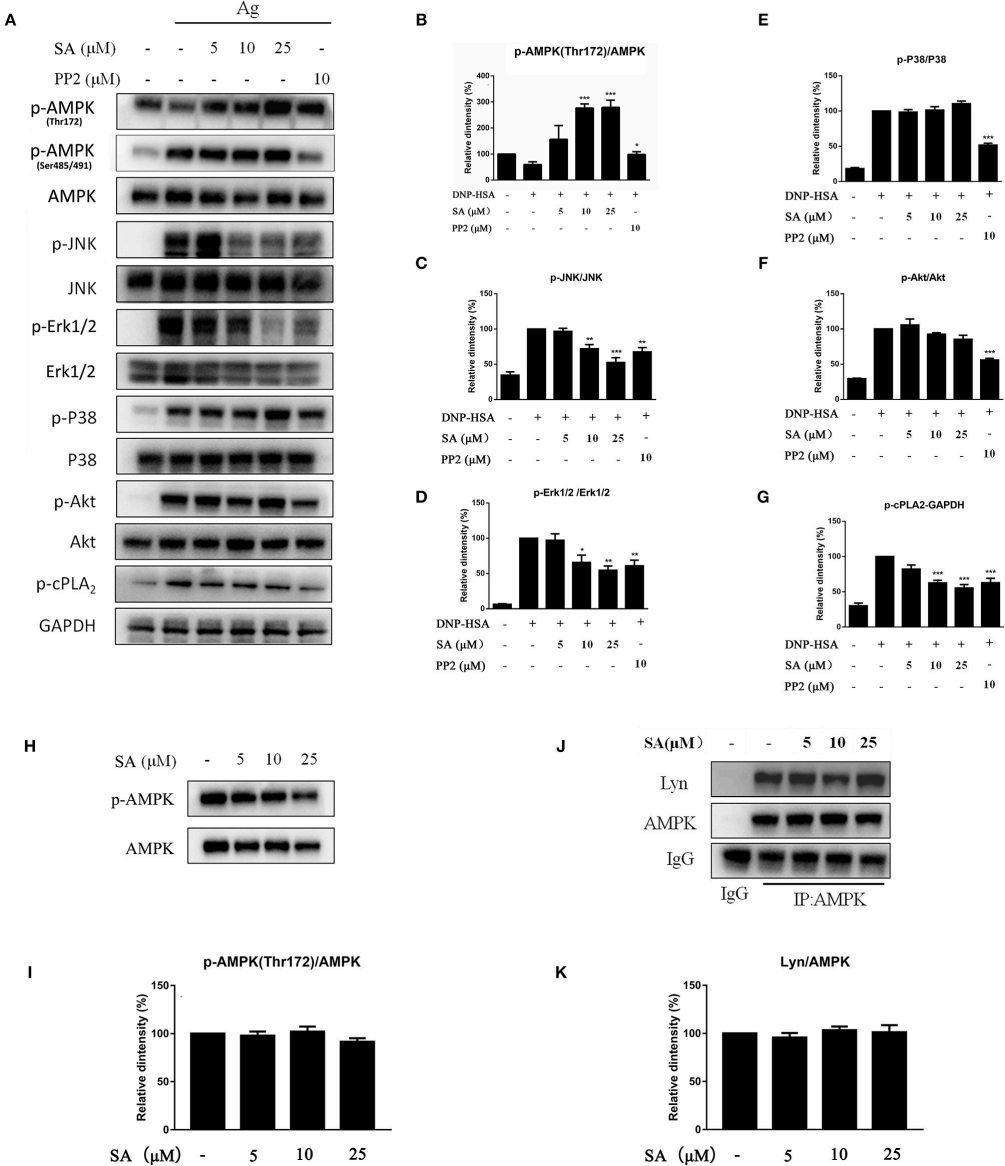
Effect of SA on AMPK and MAPK pathway. **(A)** IgE-sensitized BMMCs were treated with or without SA for 30 min and stimulated with DNP-HSA for 15 min. Then, protein lysates were analyzed for the phosphorylated and non-phosphorylated forms of AMPK, JNK, Erk1/2, P38, Akt, cPLA_2_, and GAPDH using Western blotting. **(B–G)** The related ratios of p-AMPK (Thr172)/AMPK **(B)**, p-JNK/JNK **(C)**, p-Erk1/2/Erk1/2 **(D)**, p-P38/P38 **(E)**, p-Akt/Akt **(F)**, and p-cPLA_2_/GAPDH **(G)** protein levels were determined using densitometry statistical analysis. *P < 0.05, **P < 0.01, ***P < 0.001 vs. the IgE/Ag-treated group. **(H)** BMMCs were treated with or without SA for 30 min, and then protein lysates were analyzed for p-AMPK (Thr172) and AMPK. **(I)** The related ratios of p-AMPK (Thr172)/AMPK were determined using densitometry statistical analysis. **(J)** BMMCs were treated with or without SA for 30 min. Cell lysates were collected and subjected to immunoprecipitation with antibodies specific for AMPK, followed by Western blotting using the indicated antibodies. **(K)** The related ratios of Lyn/AMPK protein levels were determined by densitometry statistical analysis. Data are represented as mean ± SEM from three experiments.

The aforementioned data indicated that SA inhibits FcεRI-mediated mast cell activation both *in vitro* and *in vivo*. We next examined whether SA affects the activation of intracellular signaling pathways by FcεRI in BMMCs. IgE-sensitized BMMCs were pretreated with SA for 30 min, followed by DNP-HSA crosslinking for 5 min or 15 min. After 5 min of DNP-HSA stimulation, SA dose-dependently inhibited the phosphorylation of Lyn (Figures 6A,E), which is a FcεRI-proximal tyrosine kinase. We next detected the downstream signaling molecules of Lyn: FcεRIβ, FcεRIγ, Syk, LAT, and PLCγ1; as expected, SA significantly suppressed their phosphorylation (Figures 6B,C,F–J). However, Fyn, another FcεRI–proximal tyrosine kinase, was not significantly changed after treatment with SA (Figures 6A,D). Consistently, SA could not change the phosphorylation of Akt (Figures 7A,F), a downstream Fyn-dependent protein (21).

MAPK signaling is distal signaling during mast cell activation and is vital for the FcεRI-mediated production of cytokines and chemokines in mast cells (6, 22, 23). As shown in Figures 7A,C–E, after 15 min of DNP-HSA stimulation, SA considerably inhibited the phosphorylation of Erk1/2 and JNK, but p-P38 remained unaltered; in addition, SA significantly inhibited p-cPLA_2_ (Figures 7A,G), which is consistent with the inhibition of LTC_4_ release (24). Moreover, SA significantly promoted AMP-activated protein kinase (AMPK) phosphorylation at Thr172 after 15 min of DNP-HSA stimulation (Figures 7A,B), while it did not alter p-AMPK (Ser485/491). In addition, SA pretreatment alone did not affect AMPK phosphorylation in non-stimulated mast cells (Figures 7H,I).

**Figure 8:**
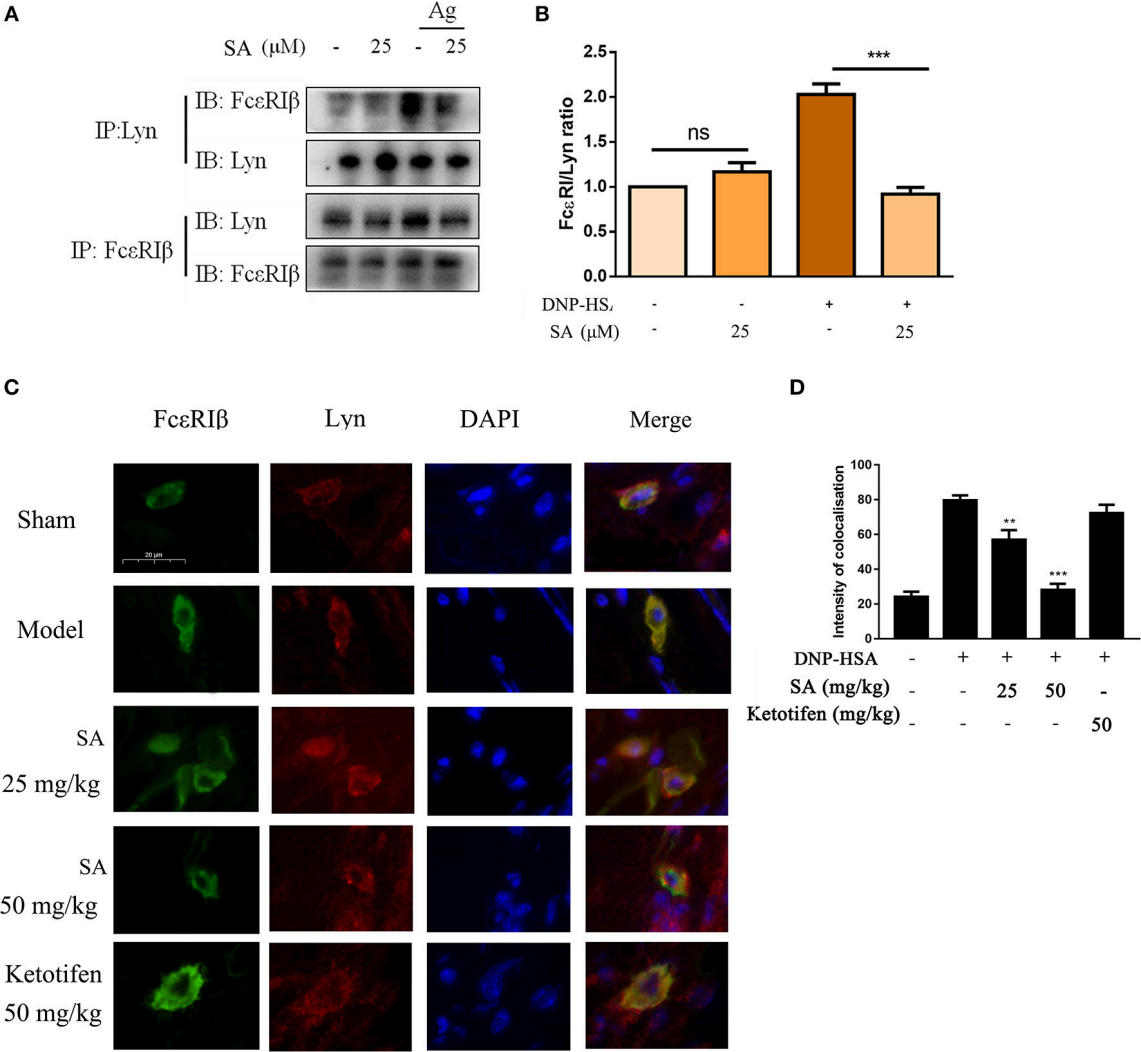
Effect of SA on the interaction between Lyn and FcεRI. **(A)** IgE-sensitized BMMCs were treated with or without SA for 30 min and then stimulated with DNP-HSA for 10 min. Cell lysates were collected and subjected to immunoprecipitation with antibodies specific for Lyn or FcεRIβ, followed by Western blotting using the indicated antibodies. **(B)** The related ratios of FcεRIβ/Lyn protein levels from anti-Lyn immunoprecipitation were determined by densitometry statistical analysis. Data are represented as mean ± SEM from three experiments. **(C)** Immunofluorescence images showing the interaction between Lyn (red) and FcεRIβ (green) in ear form PSA mice. Bars, 20 μm. **(D)** The intensity of colocalisation between Lyn and FcεRIβ, and quantified by Image-Pro Plus 6.0.

Previous report showed that Lyn-specific siRNA increase human mast cell activation (25). To further clarify the mechanism of SA in allergic response, we assessed the effects of SA on Lyn activity by using an *in vitro* enzyme system. However, SA did not inhibit Lyn kinase activation (data not shown). We then examined the effect of SA on AMPK/Lyn interaction before any FcεRI aggregation. As shown as Figures 7J,K, the interaction between AMPK and Lyn was not altered with SA. In the Lyn-related pathway, the Lyn–FcεRIβ interaction is indispensable for FcεRI-mediated mast cells (16). We examined whether SA influenced the Lyn–FcεRIβ interaction. As shown as Figures 8A,B, there was a weak binding between Lyn and FcεRIβ when mast cells were at rest status, and SA did not affect the binding between Lyn and FcεRIβ. However, allergen exposure for 10 min considerably enhanced the binding between Lyn and FcεRIβ, and SA blocked this binding, reduced it to that at mast cell rest status. On investigating whether SA blocked the binding between Lyn and FcεRIβ *in vivo* in the PSA model, we found that SA significantly suppressed the binding between Lyn and FcεRIβ after exposure to the allergen for 10 min, whereas ketotifen did not (Figures 8C,D).

## Discussion

Abietane-type diterpenes are abundant in nature and have various biological activities and pharmacological properties, such as antitumor (26), antiviral (27), antibacterial (28, 29), and anti-inflammatory (30). However, their antiallergic effect has not been reported. SA is an abietane-type diterpene from *S. dentata* Royle ex Benth. In this study, we first report that SA inhibits mast cell activation and mast cell–mediated allergic reactions.

Mast cells play a key role in allergic response in conditions such as asthma, rhinitis, and atopic dermatitis. IgE/Ag-activated mast cells activate a complex intracellular signaling pathway. Lyn and Fyn are essential for initiating FcεRI-mediated mast cell activation: Lyn mediates a pivotal signaling pathway following aggregation of FcεRIs, whereas Fyn is key in a complementary pathway for mast cell activation (31). Several studies have reported compounds that suppress IgE-mediated allergic response through the inhibition of Lyn and Fyn. For example, AZD7762 suppressed IgE-mediated mast cells and allergic responses by inhibiting the activity of Lyn and Fyn kinases (32). Elaeocarpus, which was isolated from *Elaeocarpus sylvestris* L., inhibited Fyn and Lyn phosphorylation, thereby preventing mast cell degranulation and expression of proinflammatory cytokines (33).

Unlike the aforementioned compounds, SA inhibited the phosphorylation of only Lyn and not Fyn (Figure 6A), and had no inhibitory effect on Akt (Figure 7A), a protein that belongs to Fyn-regulated compensatory signaling pathways. In experiments with RBL-2H3 cells, where Fyn gene and protein expressions are hardly detected (34), SA inhibited the degranulation and expression of proinflammatory cytokines in a dose-dependent manner (Figure 3). Therefore, speculating that SA only inhibits the Lyn-dependent pathway in IgE/Ag-induced mast cells, we investigated the effect of SA on phosphorylation of FcεRIβ, FcεRIγ, Syk, LAT, PLCγ, and MAPKs. SA significantly suppressed the phosphorylation of Syk, LAT, PLCγ, Erk1/2, and JNK, but did not affect the phosphorylation of P38.

AMPK, a regulator of energy metabolism, negatively regulates mast cell activation and anaphylaxis. AMPK activation suppressed IgE/Ag-induced mast cell activation through the inhibition of Erk and JNK phosphorylation, but did not change P38 phosphorylation (35). Therefore, we investigated the involvement of SA in IgE/Ag-induced AMPK activation. As expected, SA significantly increased AMPK phosphorylation at Thr172 after 15 min of DNP-HSA stimulation (Figure 7A), while it did not alter p-AMPK (Ser485/491). However, the AMPK phosphorylation at Thr 172 is not affected with Lyn knockdown, whereas Fyn counter-regulates the AMPK signaling pathway in mast cells (35). Thus, we suggest that the target of SA in mast cells might be simultaneously related to Lyn and AMPK.

FcεRIβ amplifies the FcεRI-mediated activation signal. The Lyn–FcεRIβ interaction is indispensable for FcεRIβ-mediated human mast cell activation (16). Lyn can pre-associate with the FcεRIβ chain before receptor crosslinking, and receptor stimulation can further increase their binding (11). In addition, AMPK binds to Lyn when mast cells are at rest, and after mast cell activation, AMPK binds to FcεRIβ (36). We hypothesized that the AMPK–FcεRIβ binding might be caused by the binding of Lyn to FcεRIβ, and that the Lyn–FcεRIβ interaction might simultaneously affect Lyn and AMPK phosphorylation. Although previous study showed that AMPK activation blocked the formation of Lyn-FcεRIβ complexes, their results are based on the AMPK phosphorylation at Ser485/491 (36). In our study, we did not observe significant effect of SA on p-AMPK (Ser 485/491) after FcεRI activation; instead, we observed that SA significantly increased AMPK phosphorylation at Thr172 after 15 min of DNP-HSA stimulation (Figure 7A). In addition, we observed that SA did not alter the interaction between AMPK and Lyn in non-stimulated mast cell (Figure 7J). And SA blocked the binding Lyn to FcεRIβ in IgE/Ag-induced mast cell both *in vitro* (Figures 8A,B) and *in vivo* (Figures 8C,D). Above all, we suggest that SA suppressed mast cell–mediated allergic response by blocking the Lyn–FcεRIβ interaction *in vitro* and *in vivo*, which is a key step in mast cell activation. Thus, SA may be a promising therapeutic agent for allergic diseases.

## Ethics Statement

All animal experiments were performed according to the Health Guidelines of the Shanghai University of Traditional Chinese Medicine, China, and protocols were approved by the Institutional Animal Ethics Committee of Shanghai University of Traditional Chinese Medicine (No. SZY201710088).

## Author Contributions

YL and JX designed the study. FQ, LZ, SL, and GM preformed the experiments. FQ and YL analyzed the data and wrote the manuscript. FG and PL provided scientific discussion.

### Conflict of Interest Statement

The authors declare that the research was conducted in the absence of any commercial or financial relationships that could be construed as a potential conflict of interest.

## Abbreviations

BSA: Bovine Serum Albumin
DMSO: Dimethyl sulfoxide
DNP-HSA: 4-dinitropheny2,l -Human serum albumin
Erk1/2: extracellular regulated protein kinases
FcεRI: the high-affinity receptor for IgE
IgE: Immunoglobulin E
ITAM: immunoreceptor tyrosine-based activatory motif
JNK: c-Jun N-terminal kinase
LAT: Linker for activation of T cell
MAPK: mitogen-activated protein kinase
P38: P38 mitogen activated protein
cPLA_2_: cytosolic phospholipase A_2_
PGD_2_: prostaglandin D_2_
PLCγ: phospholiase C-gamma
XTT: 2,3-bis (2-methoxy-4-nitro-5-sulfophenyl)−5-[(phenylamino)carbonyl]-2H-tetrazolium hydroxide
β-Hex: β-hexosaminidase
AMPK: AMP-activated protein kinase.
